# Depth segregation and diet disparity revealed by stable isotope analyses in sympatric herbivorous cichlids in Lake Tanganyika

**DOI:** 10.1186/s40851-015-0016-1

**Published:** 2015-05-14

**Authors:** Hiroki Hata, Jyunya Shibata, Koji Omori, Masanori Kohda, Michio Hori

**Affiliations:** Graduate School of Science and Engineering, Ehime University, 2-5 Bunkyo, Matsuyama, Ehime Japan; Center for Marine Environmental Studies (CMES), Ehime University, 2-5 Bunkyo, Matsuyama, Ehime Japan; Environmental Research and Management Center, Hiroshima University, 1-4-4 Kagamiyama, Higashi-Hiroshima, Hiroshima Japan; Graduate School of Science, Osaka City University, Sumiyoshi-ku, Osaka Japan; Kyoto University, Yoshida-Honmachi, Sakyo, Kyoto Japan

**Keywords:** Tanganyikan cichlid, Stable isotope, Herbivore, Ecomorph, Adaptive radiation

## Abstract

**Background:**

Lake Tanganyika in the African Great Rift Valley is known as a site of adaptive radiation in cichlid fishes. Diverse herbivorous fishes coexist on a rocky littoral of the lake. Herbivorous cichlids have acquired multiple feeding ecomorphs, including grazer, browser, scraper, and scooper, and are segregated by dietary niche. Within each ecomorph, however, multiple species apparently coexist sympatrically on a rocky slope. Previous observations of their behavior show that these cichlid species inhabit discrete depths separated by only a few meters. In this paper, using carbon (C) and nitrogen (N) stable isotope ratios as markers, we followed the nutritional uptake of cichlid fishes from periphyton in their feeding territories at various depths.

**Results:**

δ^15^N of fish muscles varied among cichlid ecomorphs; this was significantly lower in grazers than in browsers and scoopers, although δ^15^N levels in periphyton within territories did not differ among territorial species. This suggests that grazers depend more directly on primary production of periphyton, while others ingest animal matter from higher trophic levels. With respect to δ^13^C, only plankton eaters exhibited lower values, suggesting that these fishes depend on production of phytoplankton, while the others depend on production of periphyton. Irrespective of cichlid ecomorph, δ^13^C of periphyton correlated significantly with habitat depth, and decreased as habitat depth became deeper. δ^13^C in territorial fish muscles was significantly related to that of periphyton within their territories, regardless of cichlid ecomorph, which suggests that these herbivorous cichlids depend on primary production of periphyton within their territories.

**Conclusions:**

Carbon and nitrogen stable isotope ratios varied among ecomorphs and among cichlid species in the same ecomorphs sympatrically inhabiting a littoral area of Lake Tanganyika, suggesting that these cichlids are segregated by nutrient source due to varying dependency on periphyton in different ecomorphs (especially between grazers and browsers), and due to segregation of species of the same ecomorph by feeding depth, grazers and browsers in particular.

**Electronic supplementary material:**

The online version of this article (doi:10.1186/s40851-015-0016-1) contains supplementary material, which is available to authorized users.

## Background

Cichlid fish communities in Lake Tanganyika are a magnificent example of adaptive radiation, in which mulitple species rapidly evolve from a common ancestor as a consequence of their adaptation to various ecological niches. After the formation of the lake 9–12 Ma, more than 200 species have diverged from eight colonizing lineages [1-4].

In a rocky littoral of Lake Tanganyika, 17 species of herbivorous cichlids coexist [5,6]. These include 11 Tropheini, three Lamprologini, one Ectodini, one Eretmodini, and one Tilapini species (Table 1). Therefore, this herbivorous fish community has become established through repetitive adaptations to herbivory in these cichlid tribes [4].

**Table 1:** **Study species of herbivorous fishes in Lake Tanganyika and their ecomorphs based on feeding habits, territoriality, and the number of samples we collected**

**Tribe**	**Species**	**Abbreviation**	**Feeding ecomorph**	**Feeding territory**	**Number of algal farms**	**Sampling depth (m)**	**Number of fish individuals**	**Reference**
Tilapiini	*Oreochromis tanganicae*	Otan	biter	no	-	-	5	[13,52]
Ectodini	*Xenotilapia papilio*	Xpap	scooper	breeding pairs only	-	-	5	[13,14]
Eretmodini	*Eretmodus cyanostictus*	Ecya	scraper	breeding pairs only	5	2.2(1.9-2.4)	5	[53,54]
Lamprologini	*Telmatochromis temporalis*	Ttem	browser	yes	4	8.1(2.4-19.6)	5	[7,55]
Lamprologini	*Telmatochromis vittatus*	Tvit	browser	no	-		5	[56]
Lamprologini	*Variabilichromis moorii*	Vmoo	browser	yes	5	4.6(2.5-6.7)	5	[57]
Tropheini	*Interochromis loocki*	Iloo	grazer	dominant males only	3	6.8(3.1-13.0)	5	[39]
Tropheini	*Pseudosimochromis curvifrons*	Pcur	browser	dominant males only	5	1.3(1.0-2.1)	5	[7,20]
Tropheini	*Petrochromis famula*	Pfam	grazer	dominant males only	-	-	5	[19]
Tropheini	*Petrochromis fasciolatus*	Pfas	grazer	dominant males only	-	-	5	[58,59]
Tropheini	*Petrochromis macrognathus*	Pmac	grazer	yes	5	0.3(0.3-0.4)	5	[60]
Tropheini	*Petrochromis polyodon*	Ppol	grazer	yes	4	3.0(2.5-3.3)	5	[7]
Tropheini	*Petrochromis horii*	Phor	grazer	yes	3	15.2(15.0-15.7)	4	[61]
Tropheini	*Petrochromis trewavasae*	Ptre	grazer	yes	6	10.1(6.4-13.7)	5	[7]
Tropheini	*Simochromis diagramma*	Sdia	browser	no	-	-	5	[7]
Tropheini	*Tropheus moorii*	Tmoo	browser	yes	3	8.7(6.0-10.5)	5	[7,16,62]
Tropheini	*Limnotilapia dardennii*	Ldar	browser	no	-	-	5	[10]
non-cichlid	*Lamprichthys tanganicanus*	Ltan	plankton eater	no	-	-	5	[63]
non-cichlid	mixed of *Stolothrissa tanganicae* and *Limnothrissa miodon*	Kape	plankton eater	no	-	-	5	[63]

Sampling depth indicate the depth in which algal farm samples were collected, shown as in average (minimum - maximum).

Tanganyikan cichlids are unique in the richness of convergent forms that evolved in the lake and coexist in similar habitats [4]. Five tribes of the family have acquired multiple herbivorous feeding ecomorphs; specifically, grazer, browser, scooper, and scraper [7-10]. Grazers comb unicellular algae from epilithic assemblages using multiple rows of similar-sized slender teeth with fork-like tricuspid tips [11,12]. Browsers nip and nibble filamentous algae using their bicuspid teeth, which line the outermost edges of both jaws [9]. Scoopers protrude and thrust their jaws into sand, intake a small amount of sand, and then eject it from the mouth or gill-openings to filter prey [13,14]. Scrapers rub epiphyton from rock surfaces using several rows of chisel-like teeth [15]. Fishes in each feeding ecomorph exhibit distinct specialized morphologies, such as jaw structure [8,16] and intestine length [17,18], physiological abilities, such as specific digestive enzymes [17], and specialized behaviours such as cropping frequency, substratum choice for feeding [7,16] and territoriality [19,20]. How do sympatric herbivorous cichlid species specialize by feeding depth and consequent food-source segregation to enable coexistence of closely relative species with similar feeding ecomorphs?

Carbon and nitrogen stable isotope ratios were used as indicators of material flow from primary producers to herbivorous cichlids. Stable carbon isotopes are known to vary by water depth due to light intensity, photosynthetic activity and consequent dissolved CO_2_ availability differ along water depth [21-23]. This value can thus indicate the relative depth at which the carbon source of cichlid fish is produced. The dependence of cichlids on primary production can be estimated by nitrogen stable isotope ratio. The composition of algal farms and stomach contents were analyzed by an amplicon metagenomics approach in a previous study [6], and it shows that algal farm composition is varied in the axis of depth, but stomach contents are highly variable among cichlid species, even those inhabiting similar depth ranges. Stomach content analyses show directly what is ingested, but there are limitations; not all ingested material is assimilated, some food items are dissolved in the stomach more quickly than others, and stomach content reflects feeding during only the short periods immediately before capture [24-26]. Therefore, in addition to stomach content analysis, stable isotope markers that provide time-integrated information can be useful tools for determining dietary sources for each cichlid species and clarifying the basis of their dietary segregation.

On a rocky littoral in Lake Tanganyika, we observed algal farms of 10 herbivorous cichlid species, measured the water depth, and collected periphyton inside the farms. At the same time, specimens of 17 herbivorous cichlid species sympatrically inhabiting a rocky shore and three plankton-eating fishes were collected. Algal farms and fish muscles were analyzed using carbon and stable isotope analyses.

## Methods

### Sampling for stable isotope analysis

This study was performed in accordance with the Regulations on Animal Experimentation at Ehime University. Approval is not needed for experimentation on fishes under Japanese law, Act on Welfare and Management of Animals. We sampled 17 species of herbivorous cichlids from Kasenga Point (8°43′S, 31°08′E) near Mpulungu, Zambia at the southern tip of Lake Tanganyika in November 2010 using gill net (Table 1). The dorsal white muscles of fishes were excised and dried for stable isotope analyses. Periphyton samples were simultaneously collected from 10 territorial cichlid species. Each periphyton sample was collected from each territory of cichlid. We defined the territory as the place where the territory holder fed on periphyton and defended against conspecific and heterospecific herbivores [27]. Whether a site was located within or outside of a cichlid fish territory was determined by 20 min of observation immediately prior to sampling. Periphyton samples were dried for stable isotope analysis.

### Stable isotope analysis

The stable isotope ratio of nitrogen (N) is useful in trophic level analysis as the nitrogen pools of animals have δ^15^N signatures regularly enriched by a certain value (typically, 3.4‰) relative to their food sources [28]. Stable isotope ratios of carbon (C) differ strongly among terrestrial plants, phytoplankton, and benthic algae [29], and can be used as tracers of C pathways within food webs. Samples of fish muscles, benthic animals, detritus, and periphyton collected from cichlid territories were dried in an oven at 60°C for 24 h, and ground into fine powder. The fish and benthic animal samples were treated with 2:1 chloroform:methanol solution for 24 h to remove lipid [30]. The periphyton and detritus samples were treated with 1.0 N HCl for 24 h and then washed with distilled water twice to remove mineral carbon. These treated samples were dried in an oven at 60°C for 24 h, again. C and N stable isotope ratios (per mil) were measured using a continuous flow isotope ratio mass spectrometer (SerCon, LTD., ANCA-SL). Stable isotopes were measured as a delta (δ) value in units of per thousand deviations from the standards (‰) and are calculated as *δX* = [(*R*_*sample*_/*R*_*standard*_) − 1] × 10^3^, where *X* is ^15^N or ^13^C, and *R* is the ratio of the heavy (^15^N or ^13^C) to the light (^14^N or ^12^C) isotope.

### Statistical analysis for stable isotope data

We analyzed δ^15^N and δ^13^C values of fish muscles and periphyton within their feeding territories using a generalized linear mixed model (GLMM). The category of ecomorph was included as a fixed factor, and cichlid species as a nested factor. GLMM was conducted by an R package, lmerTest 2.0-3. Tukey’s post-hoc test was applied to compare mean differences between ecomorphs using the glht function in the multcomp package in R. The differences of δ^15^N and δ^13^C values of fish muscles and periphyton within their territories were analyzed using a generalized linear model (GLM) for each ecomorph using cichlid species as a fixed factor. GLM is conducted by glm function in R 3.0.2 [31]. Tukey’s post-hoc test was applied to compare mean differences between species using the glht function in the multcomp package in R. To test the effect of depth on δ^13^C and δ^15^N of algal farms, GLMM was conducted with depth as a fixed factor and cichlid species as a random factor. A GLMM was also conducted to test the effect of C and N stable isotope ratios in periphyton and cichlid ecomorphs on the isotope ratios in the muscles of territorial cichlids. Species were included as a random factor.

### Stable isotope mixing model

Probability distributions for the proportional contributions of the potential dietary sources to the diet of each cichlid species were determined using the Bayesian stable isotope mixing model (MixSIAR), using MixSIAR GUI 1.0 [32,33]. δ^15^N and δ^13^C of each cichlid species were used as mixture data, and the same values from periphyton within territories defended by each cichlid species and those of other benthic animals and detritus were used as source data, together with their C and N concentration values. Markov Chain Monte Carlo parameters were set as follows, chain length = 50,000, burn in = 25,000, thin = 25, number of chains = 3. Trace plots and the result of Gelman-Rubin, Heidelberger-Welch, and Geweke diagnostic tests were used to confirm that the model had converged [33]. Discrimination values for δ^15^N and δ^13^C were provided as 3.4 ± 1.5‰ and 0.9 ± 1.1‰ [average ± standard deviation (SD)], respectively following Cabana and Rasmussen [28] and Harvey et al. [34], but SD of δ^15^N was enlarged because the discrimination value of δ^15^N can be larger in herbivorous fishes [35,36].

## Results and discussion

### Difference in C and N stable isotope ratios among ecomorphs

**Figure 1:**
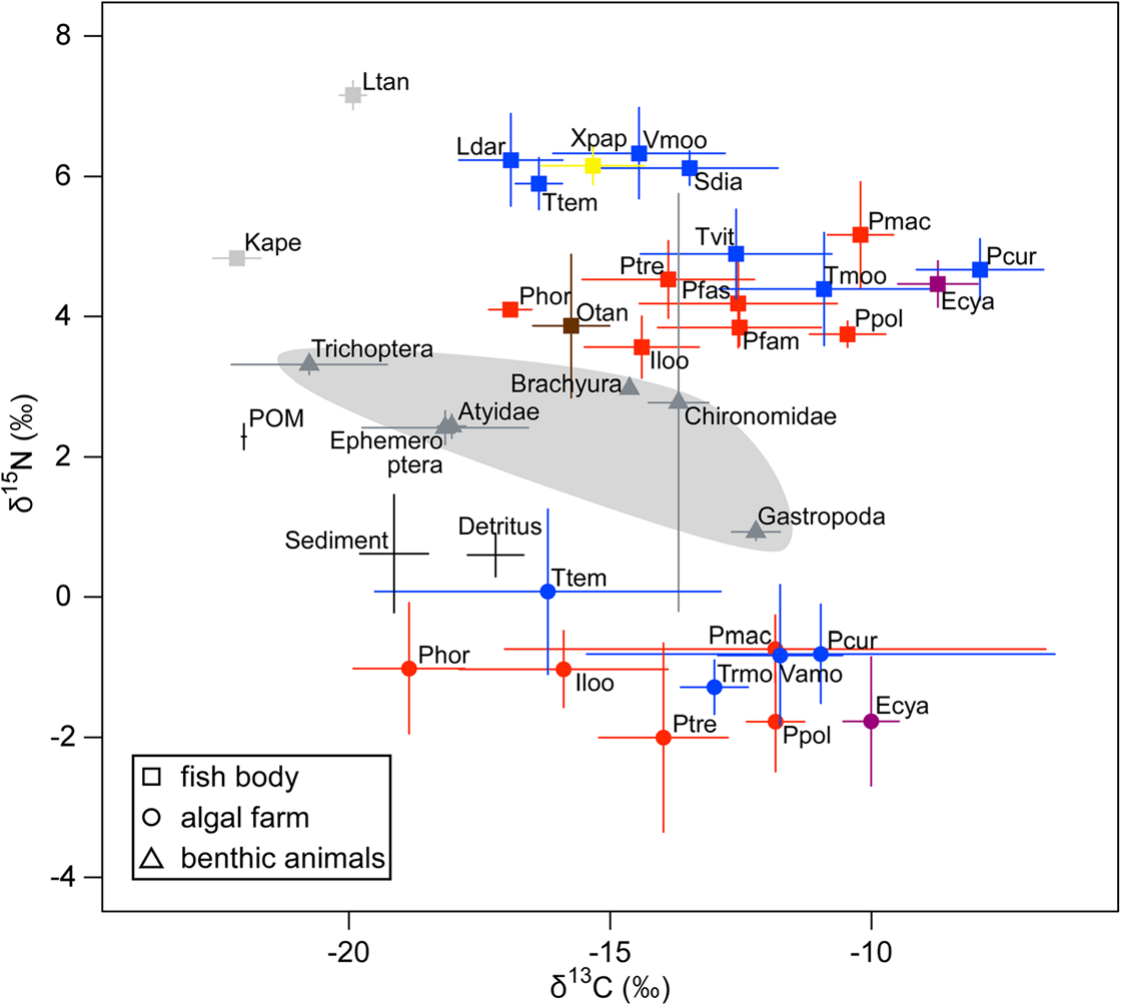
δ^13^C-δ^15^N map for herbivorous cichlids, periphytons inside their algal farms, and other potential dietary items such as benthic animals, detritus, sediment, and particulate organic matter (POM). Abbreviations of cichlid species are shown in Table 1. Square, circle, and triangle indicate samples of fish muscles, periphyton collected from each cichlid territory, and benthic animals, respectively. Red, blue, purple, and plots indicate each ecomorph, grazer, browser, and scraper respectively. Plots of benthic animals are enclosed in a grey shadow. Error bars indicate standard deviations.

δ^13^C and δ^15^N stable isotope ratios of herbivorous cichlid muscles and periphyton within their algal farms varied widely as shown in Figure 1. As a result of GLMMs, both δ^13^C and δ^15^N values of cichlid muscles were significantly different among feeding ecomorphs (Table 2). The muscle δ^15^N was significantly lower in grazer than browsers, although δ^15^N values of periphyton within territories were not different among territorial species (Tables 2 and 3), suggesting that grazers depend more directly on primary production of periphyton, while others ingest animals with higher trophic level. This result agrees with the observations in previous studies. Previous studies show that grazers comb unicellular algae and cyanobacteria from the epiphytic assemblages using brush-like jaws [11,12,37], and animals were rarely found in their stomachs [7,10,38]. On the other hand, browsers nip and nibble filamentous algae and cyanobacteria using their nail clipper-like jaws [8,16,37], and *Telmatochromis temporalis*, *Limnotilapia dardennii*, and *Simochromis diagramma* ingest ephemeropteran and dipteran larvae, and fish fry [7,10,38]. *Xenotilapia papilio*, a scooper, had a relatively high value of δ^15^N (Figure 1), partly as this fish intakes and filters sand to capture diptera and copepoda, as well as algae and cyanobacteria within sand [14,38].

**Table 2:** **Results of generalized linear mixed-model analyses to test the effect of cichlid ecomorphs on carbon and nitrogen stable isotope ratios of their muscles**

**δ** ^**13**^ **C**					
Ecomorph	Estimate	SE	DF	*t* value	*p*
Intercept (biter)	−15.75	2.69	13.0	−5.86	<0.001
browser	2.51	2.87	13.0	0.88	NS
grazer	2.76	2.87	13.0	0.96	NS
plankton eater	−5.29	3.29	13.0	−1.61	NS
scooper	0.42	3.80	13.0	0.11	NS
scraper	7.02	3.80	13.0	1.85	NS
Post-hoc test	Estimate	SE		*z* value	*p*
browser-biter	2.51	2.87		0.88	NS
grazer-biter	2.76	2.87		0.96	NS
plankton eater-biter	−5.29	3.29		−1.61	NS
scooper-biter	0.42	3.80		0.11	NS
scraper-biter	7.02	3.80		1.85	NS
grazer-browser	0.25	1.44		0.17	NS
plankton eater-browser	−7.80	2.15		−3.62	<0.01
scooper-browser	−2.10	2.87		−0.73	NS
scraper-browser	4.50	2.87		1.57	NS
plankton eater-grazer	−8.05	2.15		−3.74	<0.01
scooper-grazer	−2.34	2.87		−0.82	NS
scraper-grazer	4.26	2.87		1.48	NS
scooper-plankton eater	5.70	3.29		1.73	NS
scraper-plankton eater	12.30	3.29		3.74	<0.01
scraper-scooper	6.60	3.80		1.74	NS
**δ** ^**15**^ **N**					
Ecomorph	Estimate	SE	DF	*t* value	*p*
Intercept (biter)	3.87	0.81	13.0	4.77	<0.001
browser	1.64	0.87	13.0	1.89	NS
grazer	0.30	0.87	13.0	0.34	NS
plankton eater	2.13	0.99	13.0	2.14	NS
scooper	2.29	1.15	13.0	2.00	NS
scraper	0.60	1.15	13.0	0.52	NS
Post-hoc test	Estimate	SE		*z* value	*p*
browser-biter	1.64	0.87		1.89	NS
grazer-biter	0.30	0.87		0.34	NS
plankton eater-biter	2.13	0.99		2.14	NS
scooper-biter	2.29	1.15		2.00	NS
scraper-biter	0.60	1.15		0.52	NS
grazer-browser	−1.34	0.43		−3.10	<0.05
plankton eater-browser	0.49	0.65		0.75	NS
scooper-browser	0.65	0.87		0.75	NS
scraper-browser	−1.04	0.87		−1.20	NS
plankton eater-grazer	1.83	0.65		2.82	<0.05
scooper-grazer	1.99	0.87		2.30	NS
scraper-grazer	0.30	0.87		0.35	NS
scooper-plankton eater	0.16	0.99		0.16	NS
scraper-plankton eater	−1.53	0.99		−1.54	NS
scraper-scooper	−1.69	1.15		−1.47	NS

Species are included as a nested factor of ecomorph. SE, standard error; DF, degree of freedom; NS, not significant.

**Table 3:** **Results of the generalized linear mixed-model analysis for testing the effect of cichlid ecomorph on carbon and nitrogen stable isotope ratios of periphyton within their territories**

**δ** ^**13**^ **C**					
Ecomorph	Estimate	SE	DF	*t* value	*p*
Intercept (browser)	−13.17	1.20	6.4	−10.98	<0.001
grazer	−1.24	1.61	6.4	−0.77	NS
scraper	3.12	2.63	5.9	1.19	NS
Post-hoc test	Estimate	SE		*z* value	*p*
grazer-browser	−1.24	1.61		−0.77	NS
scraper-browser	3.12	2.63		1.19	NS
scraper-grazer	4.36	2.57		1.69	NS
**δ** ^**15**^ **N**					
Ecomorph	Estimate	SE	DF	*t* value	*p*
Intercept (browser)	−0.81	0.30	7.0	−2.70	<0.05
grazer	−0.57	0.41	7.2	−1.41	NS
scraper	−1.17	0.65	6.1	−1.80	NS
Post-hoc test	Estimate	SE		*z* value	*p*
grazer-browser	−0.57	0.41		−1.41	NS
scraper-browser	−1.17	0.65		−1.80	NS
scraper-grazer	−0.59	0.64		−0.93	NS

Species are included as a nested factor of ecomorph. SE, standard error; DF, degree of freedom; NS, not significant.

With regard to δ^13^C, plankton eaters (*Limnothrissa miodon* and *Stolothrissa tanganicae*) had significantly lower values, suggesting that these fishes depend on phytoplankton as a carbon source as δ^13^C of phytoplankton is known to be lower than that of benthic algae [29]. On the other hand, no significant difference was found in δ^13^C among the other ecomorphs, suggesting that all of the herbivorous cichlids depend on periphyton as carbon source.

### Difference among species within ecomorphs

**Figure 2:**
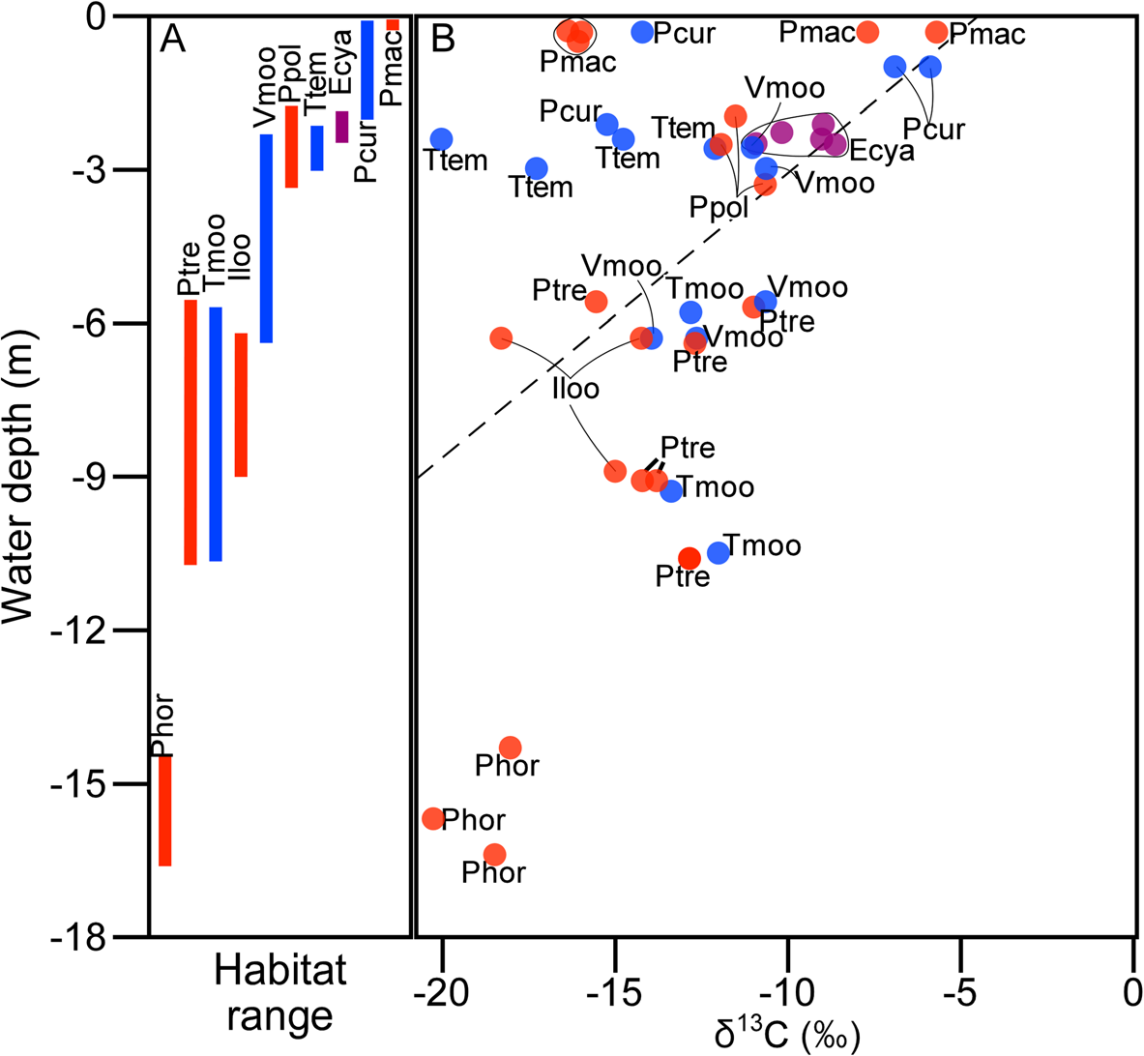
Habitat depth of each cichlid species **(A)** and relation between habitat depth and δ^13^C of periphyton **(B)**. Dotted line in B indicates the fitted line. Red, blue, and purple colors indicate cichlid ecomorph, grazer, browser, and scraper, respectively.

In both browsers and grazers, muscle δ^15^N and δ^13^C differed significantly among species (Table 4). Muscle δ^13^C of *L. dardennii* and *T. temporalis* were significantly smaller than that of the other browsers, δ^13^C of *Petrochromis horii* was smallest, and that of *Interochromis loocki*, and *P. trewavasae* were intermediate, and the values of other grazers were significantly higher (Table 4). Although δ^13^C of their algal farms were not significantly varied between species in grazers (Table 5), significant positive correlation was shown between δ^13^C of periphyton and the depth those samples were collected (Figure 2, Table 6). This tendency is due partly to the fact that relative content of δ^13^C of algae and cyanobacteria increases when growth rate/photosynthesis activity becomes higher, and when available aqueous CO_2_ decreases [39]. These herbivorous cichlids segregate their habitat depth by species in a-few-meter scale (Figure 2, [5-7,16]), and differences in habitat depth cause differences in δ^13^C of periphyton within cichlid territories.

**Table 4:** **Results of generalized linear models for testing the effect of cichlid species of each ecomorph on carbon and nitrogen stable isotope ratios of their muscles**

δ^**13**^ **C**					
browser					
		Estimate	SE	*t* value	*p*
	Intercept (Ldar)	−16.9	0.7	−25.3	<0.001
	Pcur	9.0	0.9	9.5	<0.001
	Sdia	3.4	0.9	3.6	<0.01
	Tmoo	6.0	0.9	6.3	<0.001
	Ttem	0.5	0.9	0.6	NS
	Tvit	4.3	0.9	4.6	<0.001
	Vmoo	2.5	0.9	2.6	<0.05
grazer					
		Estimate	SE	*t* value	*p*
	Intercept (Iloo)	−14.4	0.6	−25.2	<0.001
	Pfam	1.9	0.8	2.3	<0.05
	Pfas	1.8	0.8	2.3	<0.05
	Pmac	4.2	0.8	5.2	<0.001
	Ppol	3.9	0.8	4.9	<0.001
	Phor	−2.5	0.9	−2.9	<0.01
	Ptre	0.5	0.8	0.6	NS
δ^**15**^ **N**					
browser					
		Estimate	SE	*t* value	*p*
	Intercept (Ldar)	6.2	0.3	24.3	<0.001
	Pcur	−1.6	0.4	−4.3	<0.001
	Sdia	−0.1	0.4	−0.3	NS
	Tmoo	−1.8	0.4	−5.1	<0.001
	Ttem	−0.3	0.4	−0.9	NS
	Tvit	−1.3	0.4	−3.7	<0.001
	Vmoo	0.1	0.4	0.3	NS
grazer					
		Estimate	SE	*t* value	*p*
	Intercept (Iloo)	3.6	0.2	16.5	<0.001
	Pfam	0.3	0.3	0.9	NS
	Pfas	0.6	0.3	2.0	NS
	Pmac	1.6	0.3	5.3	<0.001
	Ppol	0.2	0.3	0.6	NS
	Phor	0.5	0.3	1.7	NS
	Ptre	1.0	0.3	3.2	<0.01

SE, standard error; NS, not significant.

**Table 5:** **Results of generalized linear models for testing the effect of herbivorous cichlid species of each ecomorph on carbon and nitrogen stable isotope ratios of periphyton within their territories**

**δ** ^**13**^ **C**					
browser					
	Species	Estimate	SE	*t* value	*p*
	Intercept (Pcur)	−11.6	1.3	−9.1	<0.001
	Tmoo	−1.4	2.1	−0.7	NS
	Ttem	−4.5	1.9	−2.3	<0.05
	Vmoo	−0.5	1.8	−0.3	NS
grazer					
	Species	Estimate	SE	*t* value	*p*
	Intercept (Iloo)	−15.9	1.6	−10.2	<0.001
	Pmac	3.2	2.0	1.6	NS
	Ppol	4.1	2.1	2.0	NS
	Phor	−2.9	2.2	−1.3	NS
	Ptre	2.3	1.9	1.2	NS
**δ** ^**15**^ **N**					
browser					
		Estimate	SE	*t* value	*p*
	Intercept (Pcur)	−1.0	0.4	−2.3	<0.05
	Tmoo	−0.3	0.7	−0.5	NS
	Ttem	1.0	0.6	1.7	NS
	Vmoo	−0.1	0.6	−0.2	NS
grazer					
		Estimate	SE	*t* value	*p*
	Intercept (Iloo)	−1.0	0.5	−2.0	NS
	Pmac	0.2	0.7	0.3	NS
	Ppol	−0.7	0.7	−1.1	NS
	Phor	0.0	0.7	0.0	NS
	Ptre	−1.1	0.7	−1.7	NS

SE, standard error; DF, degree of freedom; NS, not significant.

**Table 6:** **Results of the generalized linear mixed-model analysis testing the effect of habitat depth on carbon and nitrogen stable isotope ratios of algal farms**

	**Estimate**	**SE**	**DF**	***t*** **value**	***p***
δ^13^C					
(Intercept)	−11.64	0.88	8.7	−13.19	<0.001
depth	0.35	0.13	12.2	−2.69	<0.05
δ^15^N					
(Intercept)	−1.06	0.32	9.3	−3.30	<0.01
depth	0.03	0.05	12.8	−0.65	NS

Cichlid species was analyzed as a random factor. SE, standard error; DF, degree of freedom; NS, not significant.

**Figure 3:**
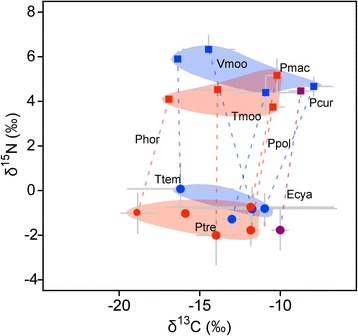
δ^13^C-δ^15^N map for herbivorous cichlids (square plots) and their algal farms (circle plots). The same species pair is connected by a broken line. Abbreviations of cichlid species are shown in Table 1. Red, blue, and purple plots indicate each ecomorph, grazer, browser, and scraper, respectively. Error bars indicate standard deviations.

δ^13^C values in muscles of territorial cichlids were also significantly affected by δ^13^C value of the periphyton within their territories, irrespective of ecomorph, although δ^15^N of muscles was not related to that of periphyton (Figure 3, Table 7). This correlation in δ^13^C suggests that these herbivorous cichlids depend on the primary production of the periphyton within their territories, especially for their carbon sources.

**Table 7:** **Results of generalized linear mixed model for testing the effect of** δ^**13**^ **C/**δ^**15**^ **N in the periphyton within territories and the effect of fish ecomorph on** δ^**13**^ **C/**δ^**15**^ **N of cichlid muscles**

	**Estimate**	**SE**	**DF**	***t*** **value**	***p***
δ^13^C					
(Intercept)	1.7	4.8	5.1	0.4	NS
δ^13^C in periphyton	1.1	0.4	5.1	3.0	<0.05
grazer in ecomorph	0.6	1.6	5.0	0.4	NS
scraper in ecomorph	0.3	2.8	5.0	0.1	NS
δ^15^N					
(Intercept)	5.8	0.6	5.0	10.0	<0.001
δ^15^N in periphyton	0.5	0.5	5.0	1.0	NS
grazer in ecomorph	−0.6	0.6	5.0	−0.9	NS
scraper in ecomorph	−0.2	1.1	5.0	−0.2	NS

Fish species are included as a random factor. SE, standard error; DF, degree of freedom.

Difference in δ^15^N implies the difference in intake of animal matters. δ^15^N of *Pseudosimochromis curvifrons*, *Simochromis diagramma*, *Tropheus moorii*, and *Telmatochromis vittatus* were significantly smaller than that of *L. dardennii* (Table 4), δ^15^N of *Petrochromis trewavasae* and *P. polydon* were higher than that of *I. loocki. L. dardennii* ingest detritus in addition to algae and cyanobacteria [10], and detritus appears to enrich δ^15^N in this cichlid by its higher δ^15^N value compared to periphyton (Figure 1). It should also be noted that Yamaoka et al. [40] suggests that *I. loocki* is a strict herbivore.

### Differences in C and N stable isotope ratios between fish muscles and periphyton within their defending algal farms

**Figure 4:**
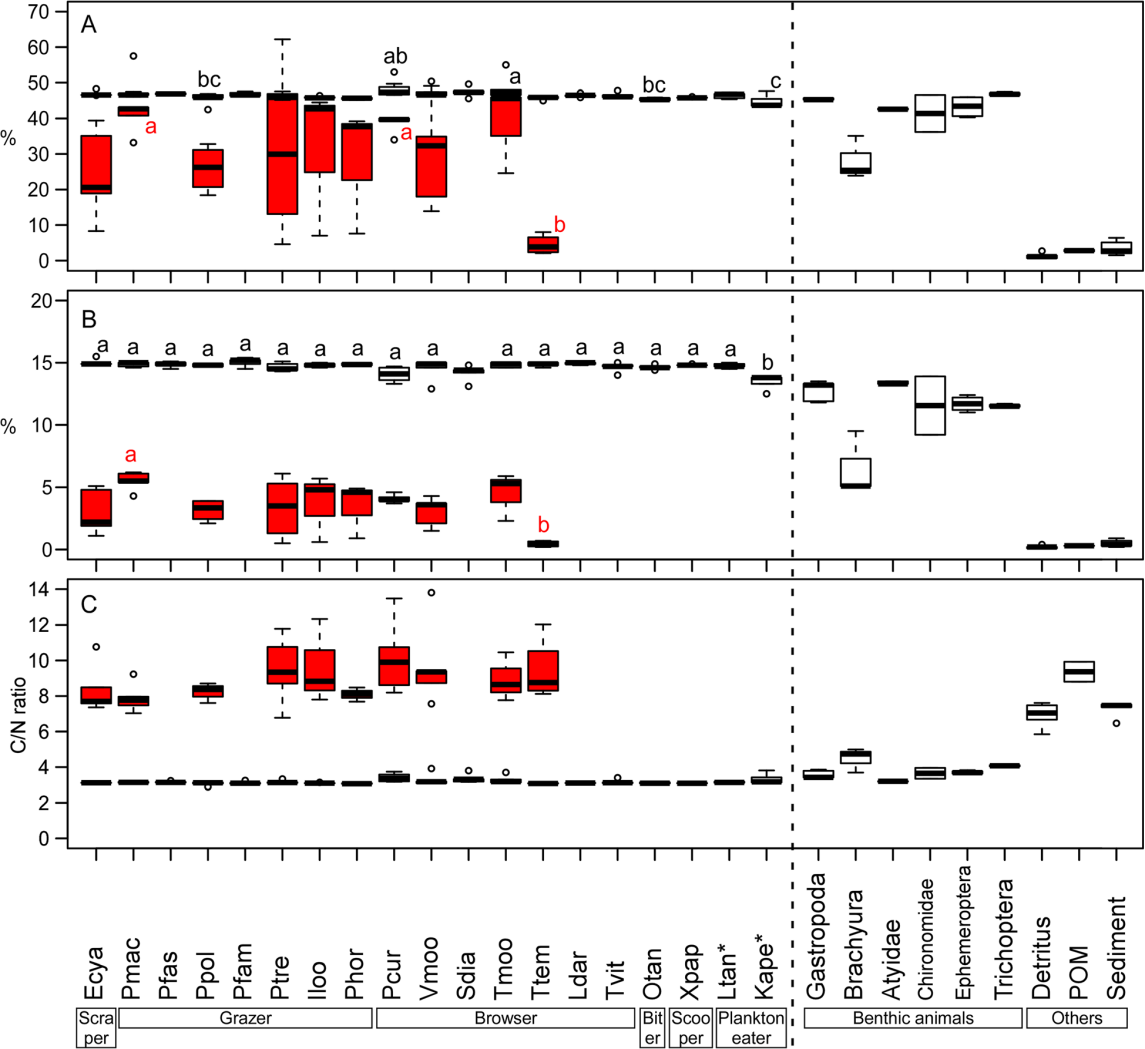
Box plots of carbon **(A)** and nitrogen **(B)** contents, C/N ratio **(C)** of fish muscles, periphyton within their territories, benthic animals, detritus, sediments, and particulate organic matter (POM) in water column. Red boxes and red letters indicate values and statistical result of periphyton. Shared letters on boxes indicate no significant differences, and pairs that do not share any letters in common were significantly different by the Tukey’s post hoc test between fish species. Species abbreviations are shown in Table 1. *denotes non-cichlid fish.

Fractions in δ^15^N between cichlid muscles and their algal farms were 5.9 ± 0.7‰ (average ± SD, *n* = 10 species) and were large differentials comparing to 3.4‰, which is the most cited value as a diet-tissue discrimination factor [41-44]. The results of our Bayesian mixed-model show that territorial herbivorous species depend mostly on periphyton and detritus within territories, both occupying 51.9–69.1% in total, except for *T. temporalis* and *V. moorii* that utilize more benthic animals (Table 8). δ^15^N of these cichlids were significantly higher, and δ^13^C were significantly smaller than those of other territorial and herbivorous cichlids (Additional file 1: Table S1). It is known, however, that the trophic-step fractionation in herbivorous fishes varies and some have relatively higher values (e.g., 4.8 ± 1.3‰ in herbivorous fishes on coral reefs) partly because of higher excretion rates in such fishes [35]. Dependency on periphyton by these cichlids may thus be an underestimate. In our system, nitrogen contents of periphytons and detritus were low (3.4 ± 1.9%, 0.2 ± 0.1%, respectively, average ± SD) and their C/N ratios were much higher (9.0 ± 1.7, 6.9 ± 0.7, respectively) than those of cichlid fishes (3.2 ± 0.2, Figure 4). Therefore, these herbivorous cichlids appear to require other nitrogen sources with high nitrogen contents and low C/N ratios, such as benthic animals, to meet their nitrogen demand. These nitrogen supplies from animal matters may partly cause the enrichment of δ^15^N in these herbivorous cichlids [45].

**Table 8:** **Predicted diet proportion of herbivorous cichlids in Lake Tanganyika from a Bayesian mixing model with** δ^**13**^ **C and** δ^**15**^ **N of each cichlid species as mixture data, those of periphyton within their territories and those of other benthic animals as source data**

**cichlid species**	**periphyton within each territory**	**detritus**	**Atyidae/Ephemeroptera**	**Trichoptera**	**Chironomidae**
*Eretmodus cyanostictus*	**43.5 ± 20.7**	24.2 ± 17.8	8.2 ± 10.1	7.5 ± 9.2	16.6 ± 13.7
*Telmatochromis temporalis*	14.6 ± 12.1	25.2 ± 16.9	**31.3 ± 17.0**	14.1 ± 11.7	14.8 ± 11.0
*Variabilichromis moorii*	**22.0 ± 15.1**	21.2 ± 16.5	20.7 ± 15.5	15.8 ± 13.2	20.2 ± 14.9
*Pseudosimochromis curvifrons*	**44.0 ± 16.7**	25.1 ± 17.2	10.8 ± 9.8	7.7 ± 7.7	12.5 ± 11.8
*Petrochromis macrognathus*	**34.3 ± 15.2**	26.6 ± 18.5	11.9 ± 10.4	9.2 ± 9.1	18.0 ± 14.1
*Petrochromis polyodon*	**46.6 ± 21.9**	22.2 ± 16.4	8.3 ± 10.5	7.3 ± 10.2	15.7 ± 13.7
*Petrochromis horii*	23.3 ± 15.0	**28.9 ± 17.7**	21.0 ± 15.3	14.6 ± 12.5	12.2 ± 11.6
*Petrochromis trewavasae*	**29.5 ± 18.0**	22.4 ± 16.9	16.5 ± 13.4	11.7 ± 11.6	20.0 ± 15.9
*Tropheus moorii*	**33.0 ± 19.6**	21.2 ± 16.4	15.0 ± 13.9	11.6 ± 12.1	19.2 ± 15.8

Analyses were conducted by MixSIAR. δ^13^C and δ^15^N of Atyidae and Ephemeroptera are quite similar (as shown in Figure 1) and cannot be distinguished. The dominant dietary items are shown in bold.

In this study, all samples for stable isotope analyses were collected a single time. It has been suggested that most primary producers have temporal variation in δ^15^N and δ^13^C because of the variation in their photosynthesis rate and in the availability of nutrients [43,46], and high temporal shifts in δ^13^C and δ^15^N in pelagic phytoplankton is also indicated in Lake Tanganyika [47]. δ^13^C and δ^15^N of herbivorous cichlids are time-integrated values reflecting their diet for a few months, and therefore, direct comparison of these values between cichlids and periphyton may have some shortcomings. This may cause the large gap in δ^15^N between herbivorous cichlids and periphyton within their territories. On the other hand, significant relation in δ^13^C between territorial cichlids and periphyton within their territories were observed. This indicate that the depth segregation among cichlids is stable as partly shown in Takeuchi et al. [5], and variation in δ^13^C along depth is relatively high comparing to the temporal variation. Further time-series sampling and analyses of periphyton and cichlid fishes will reveal the detailed habitat segregation throughout years.

### Effect of the depth segregation on cichlid diversification

Specialization at a specific depth may enhance diversification. In Lake Victoria, light environments are different by depth and cichlid species have adapted and differentiated their vision. The adapted visions are associated with the male nuptial colorations, and have led to speciation and diversification of species [48]. Further, repeated lake-level fluctuation is thought to drive diversification of Tanganyikan cichlid through the repetitive shrink and expansion of habitats [49]. One Tanganyikan cichlid, *Telmatochromis temporalis*, has diversified into two genetically-distinct ecomorphs: a large-bodied rock-living ecomorph, and a small-bodied shell-living ecomorph [50,51]. This diversification occurred repeatedly in places where rocky habitat and shell beds are adjacent. Therefore, a variant that mature in small size in original population in the rocky habitat is thought to have shifted to the shell bed when the shell bed became a suitable depth as a result of lake-level changes [51]. In this way, under stenotopic constraints for specific depths and substrata, each population undergoes local selection, and gene flow between populations living in different environments can be restricted. Further, when habitats are separated, ancestral species may be diversified into different environments and sufficiently specialized not to mix with each other after their habitats are reunified and these populations re-encounter each other.

## Conclusions

Carbon and nitrogen stable isotope ratios revealed the material flows from primary producers to herbivorous cichlids that inhabit various depths on a rocky littoral area of Lake Tanganyika. Carbon stable isotope value of primary producers was significantly correlated with the water depth at which the periphyton was collected. In the cichlids, both territorial grazers and browsers, carbon and nitrogen stable isotope values were significantly different among species, and this was caused by their habitat depth segregation. In this way, we show that multiple species of the same ecomorph living sympatrically on a rocky shore segregate not only by habitat depth but also by feeding depth. This specialization on specific depth may drive speciation and diversification, and prevent close relatives being mixed during water level fluctuations of the lake.

## Additional file

Additional file 1: Table S1. Result of Tukey’s post-hoc test of multiple comparison between cichlid species of each ecomorph on carbon and stable isotope ratios of their muscles. **Table S2.** Result of Tukey’s post-hoc test of multiple comparison between cichlid species of each ecomorph on carbon and stable isotope ratios of periphytons within their territories.

## Abbreviations

C: Carbon
DF: Degree of freedom
GLM: Generalized linear model
GLMM: Generalized linear mixed model
N: Nitrogen
NS: Not significant
POM: Particulate organic matter
SD: Standard deviation
SE: Standard error
