# Supplementary material for: Depth segregation and diet disparity revealed by stable isotope analyses in sympatric herbivorous cichlids in Lake Tanganyika
Source: Zoological Lett. 2015 May 14;1:15. doi: 10.1186/s40851-015-0016-1 (PMC4657292; doi:10.1186/s40851-015-0016-1)
Supplement: Additional file 1: Table S1. — Result of Tukey’s post-hoc test of multiple comparison between cichlid species of each ecomorph on carbon and stable isotope ratios of their muscles. Table S2. Result of Tukey’s post-hoc test of multiple comparison between cichlid species of each ecomorph on carbon and stable isotope ratios of periphytons within their territories. [file 40851_2015_16_MOESM1_ESM.docx]

# **Additional file 1 Table S1 - Result of Tukey's post-hoc test of multiple comparison between cichlid species of each ecomorph on carbon and stable isotope ratios of their muscles.**

δ^13^C

browser

Post-hoc test Estimate SE *z* value *p*

Pcur - Ldar 8.98 0.94 9.50 <0.001

Sdia - Ldar 3.42 0.94 3.62 <0.01

Tmoo - Ldar 5.99 0.94 6.35 <0.001

Ttem - Ldar 0.54 0.94 0.57 NS

Tvit - Ldar 4.31 0.94 4.56 <0.001

Vamo - Ldar 2.45 0.94 2.60 NS

Sdia - Pcur -5.56 0.94 -5.88 <0.001

Tmoo - Pcur -2.98 0.94 -3.16 <0.05

Ttem - Pcur -8.44 0.94 -8.93 <0.001

Tvit - Pcur -4.67 0.94 -4.94 <0.001

Vmoo - Pcur -6.53 0.94 -6.91 <0.001

Tmoo - Sdia 2.57 0.94 2.73 NS

Ttem - Sdia -2.88 0.94 -3.05 <0.05

Tvit - Sdia 0.89 0.94 0.94 NS

Vmoo - Sdia -0.97 0.94 -1.03 NS

Ttem - Tmoo -5.46 0.94 -5.78 <0.001

Tvit - Tmoo -1.68 0.94 -1.78 NS

Vmoo - Tmoo -3.54 0.94 -3.75 <0.01

Tvit - Ttem 3.77 0.94 3.99 <0.01

Vmoo - Ttem 1.91 0.94 2.03 NS

Vmoo - Tvit -1.86 0.94 -1.97 NS

grazer

Post-hoc test Estimate SE *z* value *p*

Pfam - Iloo 1.87 0.81 2.32 NS

Pfas - Iloo 1.85 0.81 2.29 NS

Pmac - Iloo 4.19 0.81 5.19 <0.001

Ppol - Iloo 3.94 0.81 4.88 <0.001

Phor - Iloo -2.52 0.86 -2.94 . NS

Ptre - Iloo 0.51 0.81 0.63 NS

Pfas - Pfam -0.02 0.81 -0.03 NS

Pmac - Pfam 2.32 0.81 2.87 . NS

Ppol - Pfam 2.07 0.81 2.56 NS

Phor - Pfam -4.39 0.86 -5.12 <0.001

Ptre - Pfam -1.36 0.81 -1.69 NS

Pmac - Pfas 2.34 0.81 2.90 NS

Ppol - Pfas 2.09 0.81 2.59 NS

Phor - Pfas -4.36 0.86 -5.10 <0.001

Ptre - Pfas -1.34 0.81 -1.66 NS

Ppol - Pmac -0.25 0.81 -0.31 NS

Phor - Pmac -6.70 0.86 -7.83 <0.001

Ptre - Pmac -3.68 0.81 -4.56 <0.001

Phor - Ppol -6.45 0.86 -7.54 <0.001

Ptre - Ppol -3.43 0.81 -4.25 <0.001

Ptre - Phor 3.02 0.86 3.53 <0.01

δ^15^N

browser

Post-hoc test Estimate SE *z* value *p*

Pcur - Ldar -1.57 0.36 -4.33 <0.001

Sdia - Ldar -0.12 0.36 -0.32 NS

Tmoo - Ldar -1.84 0.36 -5.09 <0.001

Ttem - Ldar -0.34 0.36 -0.93 NS

Tvit - Ldar -1.34 0.36 -3.70 <0.01

Vmoo - Ldar 0.10 0.36 0.27 NS

Sdia - Pcur 1.45 0.36 4.00 <0.01

Tmoo - Pcur -0.28 0.36 -0.76 NS

Ttem - Pcur 1.23 0.36 3.39 <0.05

Tvit - Pcur 0.23 0.36 0.62 NS

Vmoo - Pcur 1.66 0.36 4.60 <0.001

Tmoo - Sdia -1.73 0.36 -4.77 <0.001

Ttem - Sdia -0.22 0.36 -0.61 NS

Tvit - Sdia -1.22 0.36 -3.38 <0.05

Vmoo - Sdia 0.21 0.36 0.59 NS

Ttem - Tmoo 1.50 0.36 4.15 <0.001

Tvit - Tmoo 0.50 0.36 1.39 NS

Vmoo - Tmoo 1.94 0.36 5.36 <0.001

Tvit - Ttem -1.00 0.36 -2.77 NS

Vmoo - Ttem 0.44 0.36 1.20 NS

Vmoo - Tvit 1.44 0.36 3.97 <0.01

grazer

Post-hoc test Estimate SE *z* value *p*

Pfam - Iloo 0.28 0.31 0.92 NS

Pfas - Iloo 0.62 0.31 2.04 NS

Pmac - Iloo 1.60 0.31 5.26 <0.001

Ppol - Iloo 0.18 0.31 0.60 NS

Phor - Iloo 0.54 0.32 1.65 NS

Ptre - Iloo 0.97 0.31 3.17 <0.05

Pfas - Pfam 0.34 0.31 1.12 NS

Pmac - Pfam 1.32 0.31 4.34 <0.001

Ppol - Pfam -0.10 0.31 -0.32 NS

Phor - Pfam 0.26 0.32 0.79 NS

Ptre - Pfam 0.69 0.31 2.25 NS

Pmac - Pfas 0.98 0.31 3.22 <0.05

Ppol - Pfas -0.44 0.31 -1.44 NS

Phor - Pfas -0.09 0.32 -0.27 NS

Ptre - Pfas 0.34 0.31 1.13 NS

Ppol - Pmac -1.42 0.31 -4.65 <0.001

Phor - Pmac -1.07 0.32 -3.30 <0.05

Ptre - Pmac -0.64 0.31 -2.09 NS

Phor - Ppol 0.35 0.32 1.09 NS

Ptre - Ppol 0.78 0.31 2.56 NS

Ptre - Phor 0.43 0.32 1.33 NS

SE, standard error; NS, not significant.

**Table S2 - Result of Tukey's post-hoc test of multiple comparison between cichlid species of each ecomorph on carbon and stable isotope ratios of periphytons within their territories.**

δ^13^C

browser

Post-hoc test Estimate SE *z* value *p*

Tmoo - Pcur -1.36 2.10 -0.65 NS

Ttem - Pcur -4.45 1.93 -2.31 NS

Vmoo - Pcur -0.46 1.82 -0.25 NS

Ttem - Tmoo -3.08 2.19 -1.41 NS

Vmoo - Tmoo 0.90 2.10 0.43 NS

Vmoo - Ttem 3.99 1.93 2.07 NS

grazer

Post-hoc test Estimate SE *z* value *p*

Pmac - Iloo 3.18 1.98 1.61 NS

Ppol - Iloo 4.05 2.07 1.96 NS

Phor - Iloo -2.88 2.21 -1.30 NS

Ptre - Iloo 2.30 1.91 1.20 NS

Ppol - Pmac 0.87 1.82 0.48 NS

Phor - Pmac -6.06 1.98 -3.07 <0.05

Ptre - Pmac -0.88 1.64 -0.54 NS

Phor - Ppol -6.93 2.07 -3.35 <0.01

Ptre - Ppol -1.75 1.75 -1.00 NS

Ptre - Phor 5.18 1.91 2.71 NS

δ^15^N

browser

Post-hoc test Estimate SE *z* value *p*

Tmoo - Pcur -0.31 0.69 -0.46 NS

Ttem - Pcur 1.05 0.63 1.66 NS

Vmoo - Pcur -0.12 0.59 -0.21 NS

Ttem - Tmoo 1.36 0.72 1.90 NS

Vmoo - Tmoo 0.19 0.69 0.28 NS

Vmoo - Ttem -1.17 0.63 -1.86 NS

grazer

Post-hoc test Estimate SE *z* value *p*

Pmac - Iloo 0.22 0.65 0.33 NS

Ppol - Iloo -0.75 0.68 -1.10 NS

Phor - Iloo 0.01 0.73 0.02 NS

Ptre - Iloo -1.13 0.65 -1.74 NS

Ppol - Pmac -0.96 0.60 -1.61 NS

Phor - Pmac -0.20 0.65 -0.31 NS

Ptre - Pmac -1.35 0.56 -2.39 NS

Phor - Ppol 0.76 0.68 1.11 NS

Ptre - Ppol -0.39 0.60 -0.64 NS

Ptre - Phor -1.14 0.65 -1.76 NS

SE, standard error; NS, not significant.
